# MSPypeline: a python package for streamlined data analysis of mass spectrometry-based proteomics

**DOI:** 10.1093/bioadv/vbac004

**Published:** 2022-01-17

**Authors:** Simon Heming, Pauline Hansen, Artyom Vlasov, Florian Schwörer, Stephen Schaumann, Paulina Frolovaitė, Wolf-Dieter Lehmann, Jens Timmer, Marcel Schilling, Barbara Helm, Ursula Klingmüller

**Affiliations:** 1 Division Systems Biology of Signal Transduction, German Cancer Research Center (DKFZ), Heidelberg 69120, Germany; 2 Institute for Physics and CIBSS Centre for Integrative Biological Signalling Studies, University of Freiburg, Freiburg 79104, Germany

## Abstract

**Summary:**

Mass spectrometry-based proteomics is increasingly employed in biology and medicine. To generate reliable information from large datasets and ensure comparability of results, it is crucial to implement and standardize the quality control of the raw data, the data processing steps and the statistical analyses. MSPypeline provides a platform for importing MaxQuant output tables, generating quality control reports, data preprocessing including normalization and performing exploratory analyses by statistical inference plots. These standardized steps assess data quality, provide customizable figures and enable the identification of differentially expressed proteins to reach biologically relevant conclusions.

**Availability and implementation:**

The source code is available under the MIT license at https://github.com/siheming/mspypeline with documentation at https://mspypeline.readthedocs.io. Benchmark mass spectrometry data are available on ProteomeXchange (PXD025792).

**Supplementary information:**

Supplementary data are available at *Bioinformatics Advances* online.

## 1 Introduction

Mass spectrometry (MS)-based proteomics is, to date, the most comprehensive approach for quantitative profiling of proteins in a great variety of biological and clinical samples. However, regardless of sample complexity, unbiased investigation of proteomic alterations in organisms is intrinsically challenging, requiring the standardization of operational procedures in different yet interconnected areas like biochemistry, MS and bioinformatics. The latter composes a particular bottleneck as many sequential steps and a multitude of parameters are required in a bioinformatics workflow that renders them challenging to document and, as a consequence, limits reproducibility. Even minor changes to an analysis workflow can significantly affect the final results.

Ready-to-use tools, such as the MaxQuant-associated Perseus (Tyanova *et al.*, 2016), can be applied to analyze a wide variety of proteomic data. However, since the specific software settings are not stored, it is very difficult to reproduce previously obtained results. Furthermore, Perseus does not support the automation of data quality assessment or the reproducible production of high-quality figures. Several open-source packages are distributed by the Bioconductor repository (www.bioconductor.org), aiming to align and standardize the first steps of proteome data analysis and to provide statistical functionalities for relative label-free quantification of proteins. Amongst the most common packages, MSstats (Choi *et al.*, 2014), MSnbase (Gatto *et al.*, 2021), DEqMS (Zhu *et al.*, 2020) and obaDIA (Yan *et al.*, 2021) provide statistical models to derive differential protein abundances and feature graphical interfaces. However, these available applications do not support the creation of all-in-one reproducible workflows to analyze quantitative proteome data. For example, they lack opportunities for the generation of quality control (QC) reports, for the functional annotation of proteins, or the visualization of pathways/groups of proteins of interest. For the stand-alone generation of QC reports, several packages are available at Bioconductor, such as proteoQC and qcmetrics. Likewise, individual packages can be found that support the functional annotation of proteins and differential analysis, e.g. topGO and clusterProfiler. Therefore, a unified pipeline that enables the standardized and comprehensive analysis of label-free proteomics data is missing.

To address these issues, here we introduce MSPypeline. This user-friendly, all-in-one python-based proteomics pipeline integrates a set of tools, allowing the seemly and standardized preprocessing and downstream analysis of label-free data acquired in data-dependent acquisition (DDA) mode. It supports the automatic creation of QC reports, offers different normalization strategies, the functional annotation of proteins, and the visualization of proteins of interest, providing an exciting tool to analyze complex datasets. Moreover, MSPypeline offers the user the advantage of saving the exact software versions and parameters, guaranteeing reproducibility of results regardless of the computing environments.

## 2 The MSPypeline package

MSPypeline is a programing package written in Python 3 (available for 3.7 or 3.8) and uses multiple standard packages for scientific computing (pandas, numpy, sklearn and matplotlib). The recommended installation is via Conda. An intuitive and concise graphical user interface offers researchers unfamiliar with programing or data analysis the opportunity to explore and visualize their data independently and in a time-effective manner. For advanced users, MSPypeline has two additional entry points, the python module and the command line. Currently, the MSPypeline package supports the analysis of label-free shotgun proteomics data analyzed by the MaxQuant software, i.e. aggregated protein intensities after feature detection and quantification of raw MS spectra; however, the internal BaseReader class can be subclassed, allowing other data inputs, thus making the package as extensible as possible. MSPypeline builds a tree-structured analysis design (Supplementary Material) to investigate the data at distinct levels, such as cell lines, treatments or patients based on the sample names.

Several analysis methods require the determination of whether a protein can be compared between two groups. In MS data, proteins are frequently not detected at random in some samples. Yet, to ensure appropriate data analyses, the protein has to be detected (intensity >0) in a sufficient number of samples per group. MSPypeline defines the required number of samples in which the respective protein has to be detected by a sigmoidal threshold function starting at 100% for up to three samples and relaxing to 50% for 12 or more samples. Based on this threshold, there are four potential scenarios of categorizing the protein: the protein can be compared between groups A and B if it is detected above the threshold in A and B, it is unique in A if it is above threshold in A and utterly absent in B, or vice versa, and it is not considered if it is below threshold in A and B.

**Fig. 1.**
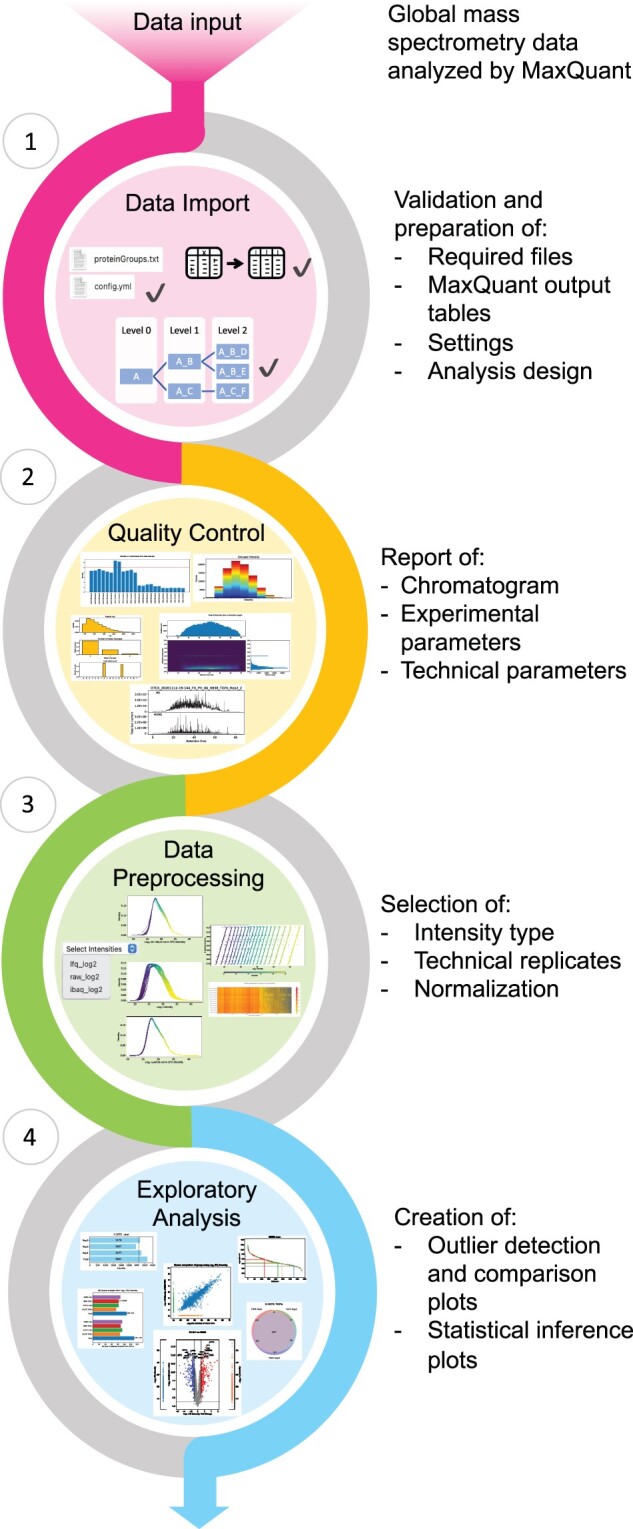
Workflow of MSPypeline. MSPypeline features a precisely structured workflow that starts with a QC report of the data, followed by the assessment and choice of data preprocessing operations to finally allow optimal exploratory analyses.

By automating the calculation and generation of versatile figures, MSPypeline performs comprehensive and conclusive data analyses within minutes. Simultaneously, the advanced user may interact closer with MSPypeline to perform advanced analysis exploiting the plethora of customization options recorded to ensure reproducibility. Although there is a logic flow linking the four different steps of analysis (Fig. 1), each step can be performed separately, making the personalization of different analyses possible.

It is worth noting that thresholding is important for the Venn group diagrams, the relative standard deviation graph, the group comparison scatter plot and the volcano plot.

The workflow for MSPypeline consists of the following steps:

1. Data import—data are loaded, converted to the required format and filtered.
2. QC—a comprehensive QC report is generated to investigate technical and biological parameters at a glance for all samples included in a given experiment.
3. Data preprocessing—tools to check normalization schemes produce plots to help to decide among five default normalization strategies (Table 1) applicable to raw, LFQ (Cox *et al.*, 2014) or iBAQ (Schwanhäusser *et al.*, 2011) intensities.
4. Exploratory analysis—descriptive and/or comparative analyses are performed on the preprocessed data allowing biologically relevant conclusions through differential expression analysis and hypothesis testing (Table 2). Visualization tools make the exploration of the results possible and include visualization by bar plots, Venn diagrams, volcano plots showing differentially regulated and unique proteins, rank plots and principal component analysis plots. All resulting plots are saved as PDF files, alongside CSV files containing the plotted data.

**Table 1.** Normalization options in MSPypeline

Normalization	Abbreviation	Description
No normalization	None	Data are not normalized.
		
Median normalization	median_norm	For each sample, the median protein intensity is calculated. The mean of all sample-wise medians is calculated and subtracted from each sample median. This correction factor is then subtracted from each protein intensity.
		
Quantile normalization with missing value handling	quantile_norm_missing_handled	Quantile normalization: for each sample, proteins are ranked after their intensity value. The mean protein intensity per quantile across all samples is calculated and assigned to every protein of each sample. The data are rearranged to the original order of the intensity values for each sample.
		Missing value handling: during normalization, missing values (protein int =0) are interpolated by sampling from the same distribution as the input distribution. After normalization, missing values are restored.
		
Tail robust quantile normalization	trqn	An offsetting factor is calculated by taking the sample-wise mean and is subtracted from each protein of the respective sample. Quantile normalization (see above) is applied, and the respective offset value is added back to each protein of the sample (Brombacher *et al.*, 2020).
		
Tail robust quantile normalization with missing value handling	trqn_missing_handled	Tail robust quantile normalization (see above) is applied with missing value handling (see above).
		
Tail robust median normalization	trmn	The sample-wise mean protein intensity is calculated and used as an offset to be subtracted from each protein of the respective sample. Median normalization (see above) is applied, and the respective offset value is added back to each protein of the sample.

**Table 2.** Default analysis options in MSPypeline

Analysis	Question	Type of plot	Analysis based on	Comparison between	Additional comments
Detection counts	How many proteins were detected how frequently in the samples of a group?	Bar diagram showing how often proteins are detected in the samples of each group.	Protein counts	Groups of the selected level	The total number of detected proteins in this group is indicated.
					
Number of detected proteins	How many proteins were detected in each of my samples and in total for each group?	Bar diagram showing the number of detected proteins per sample and the total number of detected proteins per group.	Protein counts	Groups of the selected level	The average number of detected proteins per group is indicated as a gray dashed line.
					
Venn diagrams	How large is the intersection of detected proteins of my samples in each group? How many proteins are uniquely detected in a sample?	Venn diagram showing the detected proteins for each sample of a group.	Protein counts	Samples within a group of the selected level	A classic Venn diagram shows the intersections with colored circles (≤3 samples), and a bar Venn diagram shows the size of each intersection as a bar with a combination matrix below identifying the intersections (≤6 samples).
					
Group diagrams	How large is the intersection of detected proteins between different groups? How many proteins are uniquely detected in a group?	Venn diagrams showing the number of detected proteins shared and uniquely detected in groups of the same level.	Protein counts, including thresholding	Groups of the selected level	A classic Venn diagram (≤3 samples) and a bar Venn diagram (≤6 samples).
					
PCA overview	How similar are the protein intensity values of my samples? Do samples cluster together?	Scatter plot showing the first two dimensions of a principal component analysis (PCA). PCA is performed using intensities of proteins detected in all samples.	Protein intensities	Groups of the selected level	Each group of the selected level is colored differently.
					
Intensity histogram	Do my samples show the same intensity profile? How does the intensity profile of my samples look? How similar are the intensity profiles?	Histogram showing binned protein intensities per sample. The samples of a group are presented in one graph.	Protein intensities	Samples within a group of the selected level	The mean intensity of the samples of a group is shown as a gray dashed line.
					
Relative SD	What is the relative standard deviation (SD) of the samples of a group?	Scatter plot and correlation heatmap showing the relative SD of proteins against the mean intensity of the corresponding protein.	Protein intensities, including thresholding	Samples within a group of the selected level	Lines drawn in different shades of blue indicate 10%, 20% and 30% relative SD. The number of proteins with a relative SD below these values is indicated.
					
Scatter replicates	How well do the overall protein intensities of the samples of each group correlate?	Scatter plot and correlation heatmap showing protein intensities of one sample versus another sample. Unique proteins per sample are shown at the left and bottom side of the scatter plot.	Protein intensities	Samples within a group of the selected level	Pearson’s correlation coefficient *r*^2^ is calculated for each comparison.
					
Experiment comparison	How well do the overall protein intensities of different groups correlate?	Scatter plot and correlation heatmap showing the group-averaged protein intensities of one group versus another group. Unique proteins per group are shown at the left and bottom side of the scatter plot.	Protein intensities, including thresholding	Groups of the selected level	Pearson’s correlation coefficient *r*^2^ is calculated for each comparison.
					
Rank	Where do my proteins of interest rank in intensity compared to all other proteins?	Rank plot depicting the protein intensity against the rank of the protein. The highest intensity accounts for rank 0%, the lowest for rank 100%.	Protein intensities	Groups of the selected level	If a protein is part of a selected pathway, it is presented in color. The median rank and the number of detected proteins of the selected pathways are shown.
Pathway analysis	What is the intensity of my proteins of interest, and is it significantly different in one group versus the other?	Scatter plot for each protein of the selected pathway, showing the protein intensity for all groups of the selected level.	Protein intensities	Groups of the selected level	*P*-values are calculated based on an independent *t*-test if protein counts are above threshold.
					
GO analysis	Are the proteins of a group enriched for the selected GO terms?	Bar chart showing the number of detected proteins from the selected GO terms that are found in each group of the selected level.	Protein counts	Groups of the selected level	*P*-values shown at the end of a bar indicate the calculated significance based on the one-tailed Fisher exact test. The total number of detected proteins of the selected GO term and the number of entries in the GO term list are shown.
Volcano plot (R)	Which proteins are significantly higher or lower in intensity comparing two groups? Which proteins are detected only in one group and not in the other?	Volcano plot displaying -log_10_(*P*-value) versus log_2_ fold change comparing protein intensities of two groups. Intensities of the unique proteins are shown on each side of the plot.	Protein intensities, including thresholding	Groups of the selected level	The *P*-value (focus on affected pathways and processes) and adjusted *P*-value (Benjamini + Hochberg, focus on regulated proteins) are determined using the R limma package. Calculations are corrected for the intensity–variance relationship. Either the 10 most significant proteins or the proteins of the selected pathways are annotated.

## 3 Results

To validate and visualize the functionalities of MSPypeline, a label-free DDA experiment was performed to generate a benchmark dataset deployed in the documentation for a demonstrative analysis. It serves as the built-in dataset of the software (Supplementary Material). The original MS raw data files and the MaxQuant search result files are available on the ProteomeXchange consortium via PRIDE (Deutsch *et al.*, 2020) repository (dataset identifier PXD025792). All input and output files from the benchmark dataset are wrapped with the MSPypeline release.

By providing automation and standardization of the downstream steps in the analysis of label-free proteome data, MSPypeline minimizes time-consuming and error-prone manual tasks. Moreover, new users can get started faster in analyzing proteomics datasets through the available graphical user interface because it is unnecessary to familiarize themselves with a complex analysis environment. Because MSPypeline offers the possibility of step-wise extensions, an additional advantage of this package is the possibility to link, in the future, more building blocks to its core, providing the possibility for extension while retaining the basic functionalities. Thus, MSPypeline can be easily adapted to the output of other search tools, such as Proteome Discoverer (Thermo Fisher Scientific) and OpenMS (Pfeuffer *et al.*, 2017). Similarly, MSPypeline can be adapted to analyze label-based data, e.g. stable isotope labeling by amino acids in cell culture, tandem mass tag or data-independent acquisition datasets.

## 4 Conclusions

The modular structure of MSPypeline allows it to be readily extended to meet the needs of future developments of technology. Standardization and reproducibility are ensured by automatically logging all analysis settings and saving them to a separate configuration file. Thus, MSPypeline provides a platform that supports users with their proteomics data analysis by providing insight into data quality, offering parameter adaptation when needed and generating custom figures with guaranteed reproducibility. The reliability of differential expression analysis can be improved, and the testing of biologically relevant hypotheses is fostered.

## Supplementary Material

vbac004_Supplementary_Data
