# Supplementary material for: MSPypeline: a python package for streamlined data analysis of mass spectrometry-based proteomics
Source: Bioinform Adv. 2022 Jan 17;2(1):vbac004. doi: 10.1093/bioadv/vbac004 (PMC9710650; doi:10.1093/bioadv/vbac004)
Supplement: vbac004_Supplementary_Data [file vbac004_supplementary_data.pdf]

Supplementary data

MSPypeline, a python package for streamlined data analysis of mass spectromety-based proteomics

Simon Heming [1], Pauline Hansen [1], Artyom Vlasov [1], Florian Schwörer [1], Stephen Schaumann [2], Paulina Frolovaitė [1], Wolf-Dieter Lehmann [1], Jens Timmer [2], Marcel Schilling [1], Barbara Helm [1] and Ursula Klingmüller [1]

[1] Division Systems Biology of Signal Transduction, German Cancer Research Center (DKFZ), Heidelberg, 69120, Germany and
[2] Institute for Physics and BIOSS Centre for Biological Signalling Studies, University of Freiburg, Freiburg, 79104, Germany.

1 Material and Methods

1.1 Cell Culture

The human non-small cell lung cancer (NSCLC) cell lines H838 (CRL-5844) and H1975 (CRL-5908) (Figure 1, Level 0) were obtained from ATCC and cultivated in Dulbecco's Modified Eagle's Medium (DMEM) (Lonza Bioscience, BE12-604F), supplemented with 10 % (v/v) fetal calf serum (FCS) (Thermo Fisher Scientific, 10270106) and 1 % (v/v) penicillin/streptomycin (P/S) (Thermo Fisher Scientific, 15140122). Cell cultures were maintained at 37 °C at 5 % CO_2 and 95 % relative humidity and passaged every four to five days to a maximum of 25 passages.

1.2 Cell Stimulation and Lysis

To generate a benchmark data set for analysis with *MSPypeline*, six cell culture dishes for each of the two NSCLC cell lines were seeded at 1.2×10^6 cells per 10 cm dish and cultivated in 10 ml DMEM without phenol red (Lonza, BE12-917F), supplemented with 10 % (v/v) FCS, 1 % (v/v) P/S and 2 mM L-glutamine (Thermo Fisher Scientific, 25030024). After three days of cultivation and an approximate confluency level of 90 % cells were washed twice with 10 ml Dulbecco's phosphate-buffered saline (DPBS) (pH 7.2 to 7.8) and subsequently incubated for 24 h in growth-factor depleted cultivation medium (phenol red-free DMEM (Biozym, 880019) supplemented with 1 mg bovine serum albumin (BSA) (Merck, A9418-10G), 2 mM L-glutamine and 1 % (v/v) P/S). Three dishes were stimulated with 2 ng μl^{-1} recombinant human tumor growth factor β (TGFβ) (R&D Systems, 240-B-010) for 24 h at 37 °C while three dishes were left unstimulated (Figure 1, Level 1). The experiment was thus performed in biological triplicates (Figure 1, Level 2).
For lysis, cells were washed three times with 10 ml DPBS. The supernatant was removed and cells were lysed in 900 µl of SDS-containing whole cell lysis buffer (1 % (v/v) Nonidet P-40 Substitute (Roche, 11754599001), 8 % (v/v) SDS (SERVA, 20767.03), 100 mM Tris pH 7.4 (Merck, T1503), 300 mM NaCl (Thermo Fisher Scientific, 10735921), 2 mM ethylenediaminetetraacetic acid (EDTA) pH 8.0 (AppliChem, APA3145), 0.2 % (w/v) sodium deoxycholate (SERVA, 18330.02), 2 mM Na_3VO_4, 10 mM NaF, 0.2 mg μl^{-1} 4-(2-aminoethyl) benzenesulfonyl fluoride hydrochloride

(AEBSF), 2 µg µl^{-1} aprotinin (AP) (all from Merck, S6508, S7920, A8456, A1153) in LC/MS grade H_2O (Thermo Fisher Scientific, 10728098).
Cell lysates were subsequently sonicated on ice (30 s, 75 % amplitude, 0.1 s on, 0.5 s off) and sonicated lysates were centrifuged for 10 min at 4 °C and 14000 G. The supernatant was transferred to new Eppendorf tubes and kept on ice. To ensure recovery of insoluble proteins, pellets were suspended in 8 M urea buffer (Urea (Merck, U5378), 40 mM Tris-HCl (VWR, 30024.290) pH 7.6, 0.1 mg ml^{-1} AEBSF, 1 µg ml^{-1} AP, 2x PhosStop (Merck, 4906845001) in LC/MS grade H_2O). Samples were sonicated once more, centrifuged and the supernatant was collected in a new Eppendorf tube. The final lysate of a sample comprised 1:1 (v/v) pooled combination of supernatant (SDS-containing whole cell lysis buffer) and the supernatant of the pellet (8 M urea) that was stored at −80 °C until further processing.

1.3 Mass Spectrometry Sample Preparation

The protein concentration of the cell lysates was measured using the Pierce BCA Protein Assay Kit (Thermo Fisher Scientific, 23225) following the manufacturers' instructions. Sample preparation for LS-MS analysis was performed following an adapted version of the single-pot, solid-phase enhanced sample-preparation (SP3) protocol (*1*) using 20 µg of protein. Protein disulfide bonds were reduced by adding 40 mM tris(2-carboxyethyl)phosphin (TCEP) (Merck, C4706) and alkylated with 160 mM 2-chloroacetamide (CAA) (Merck, C0267). Samples were incubated for 1 h at 37 °C in the T100 Thermal Cycler (Bio-Rad) and subsequently, the corresponding amount of hydrophobic and hydrophilic bead preparation (Merck, 45152105050250, 65152105050250; 1:1 mixture) was added. To initiate bead-to-protein binding, samples were complemented with ethanol (Merck, 1009831000) to a final concentration of 50 % (v/v) and incubated at room temperature for 10 min at 1000 G. Samples were washed three times with 80 % (v/v) ethanol in LC/MS grade H_2O and then incubated for 16 h in digestion buffer at 37 °C at 1000 G (100 mM triethylammonium bicarbonate (TEAB) (Merck, 7408), Trypsin Gold (10:1 protein: trypsin, Promega, V5280) in LC/MS grade H_2O after 30 s of sonication in 65 °C water bath. Following protein digestion, samples were separated from the beads by holding the sample tubes on a magnet for bead attraction and transferring the supernatant to new tubes. To ensure purification of the digest from any beads, this step was repeated, the recovered peptides were transferred to low protein-binding microcentrifuge tubes (Nerbe plus, 04-212-3500), lyophilized using SpeedDry Vacuum Concentrator (CHRIST, RVC 2-18 CDplus), and stored at −20 °C.

1.3.1 Mass Spectrometry Measurement and Analysis by MaxQuant

Nano-flow liquid chromatography MS/MS analysis was performed by coupling an EASY-nLC 1200 to an Orbitrap Exploris 480 MS (both from Thermo Fisher Scientific). Lyophilized samples were reconstituted in 15 µl loading buffer (0.1 % (v/v) formic acid (FA), 2 % (v/v) acetonitrile (ACN) in LC/MS grade H_2O) and 2.5 µl were injected for each analysis. Technichal replicates (Figure 1, Level 3) were established by injecting 2.5 µl from one sample two times. All 24 samples were injected and measured in a randomized manner. Peptides were delivered to an analytical column (100 µm × 30 cm, packed in-house with Reprosil-Pur 120 C18-AQ, 1.9 µm resin (Dr. Maisch)) at a flow rate of 3 µl min^{-1} in 100 % solvent A (0.1 % (v/v) FA in LC/MS grade H_2O). After loading, peptides were separated using an 84 min gradient from 2 % to 98 % of solvent B (0.1 % (v/v) FA, 80 % (v/v) ACN in LC/MS grade H_2O) at 350 nl min^{-1} flow rate. The Orbitrap Exploris 480 was operated in data-dependent mode, automatically switching between MS and MS2. Full scan MS spectra were acquired in the Orbitrap at 60,000 (m/z 200) resolution after accumulation to a target

value of 3,000,000. Tandem mass spectra were generated for up to 20 peptide precursors in the Orbitrap (isolation window 1.0 m/z) for fragmentation using higher-energy collisional dissociation at a normalized collision energy of 30 % and a resolution of 15,000 with a target value of 100,000 charges after accumulation for a maximum of 22 ms. Raw MS spectra were processed by MaxQuant (version 1.6.3.3) for peak detection and quantification. MS/MS spectra were searched against the Uniprot human reference proteome database (downloaded on October 14th, 2020) by Andromeda search engine enabling contaminant detection and the detection of reversed versions of all sequences with the following search parameters: Carbamidomethylation of cysteine residues as fixed modification and acetyl (Protein N-term), oxidation (M) as variable modifications. Trypsin/P was specified as the proteolytic enzyme with up to three missed cleavages allowed. The mass accuracy of the precursor ions was determined by the time-dependent recalibration algorithm of MaxQuant. The maximum false discovery rate for proteins and peptides was 0.01 and a minimum peptide length of eight amino acids was required. As for the rest of the settings, they correspond to the default configuration of MaxQuant and the calculation of LFQ intensities (including normalization) was selected.

1.4 Analysis by ***MSPypeline***

The following output tables that were created by MaxQuant were used for *MSPypeline*:

- **proteinGroups.txt**
- petides.txt
- parameters.txt
- summary.txt
- msScans.txt
- msmsScans.txt
- evidence.txt

The mass spectrometry proteomics data were deposited to the ProteomeXchange Consortium via the PRIDE (*2*) partner repository with the dataset identifier PXD025792.

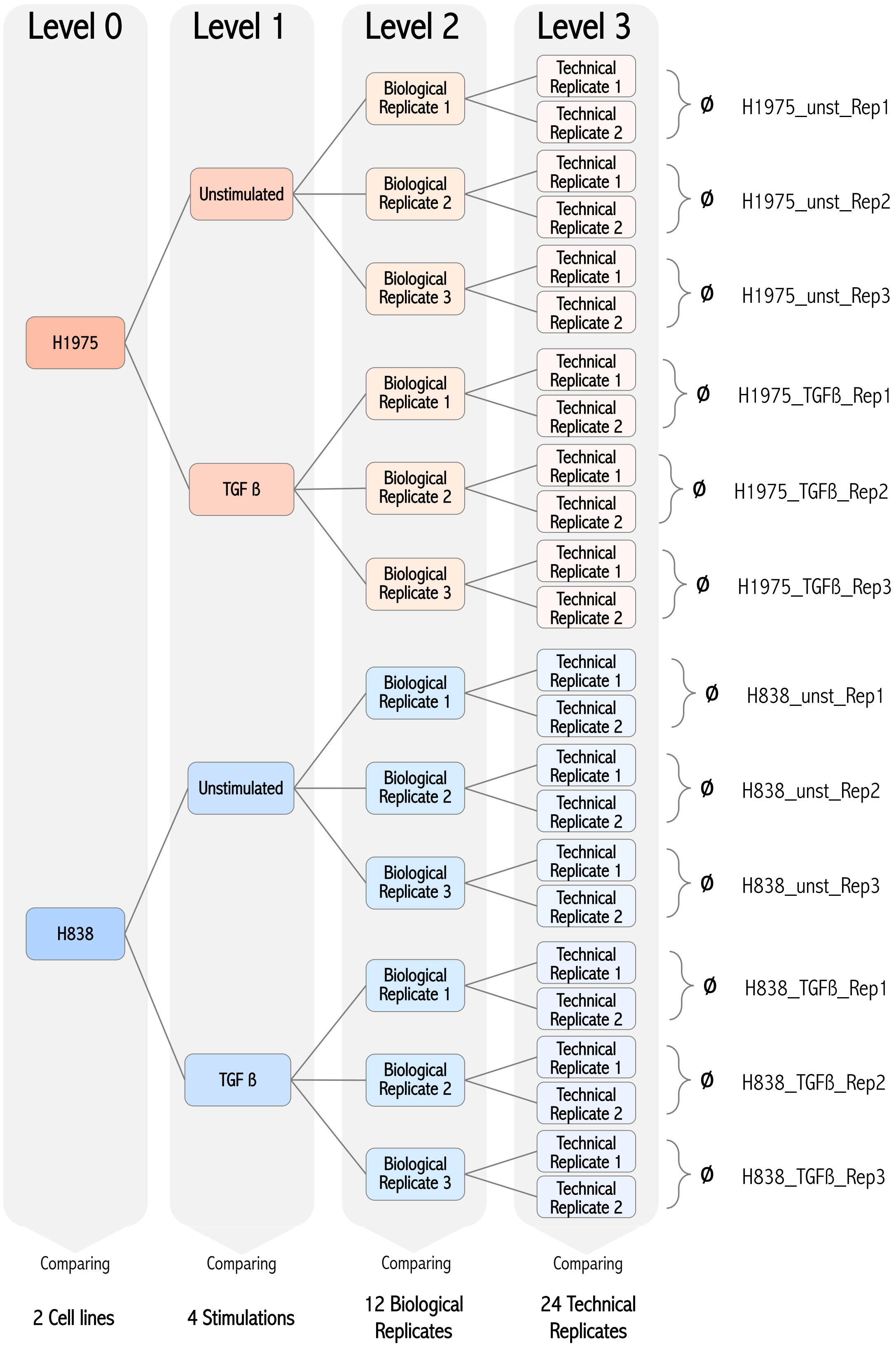
Level 0
Level 1
Level 2
Level 3
H1975
Unstimulated
TGF ß
H838
Unstimulated
TGF ß
Biological Replicate 1
Biological Replicate 2
Biological Replicate 3
Technical Replicate 1
Technical Replicate 2
Ø H1975_unst_Rep1
Ø H1975_unst_Rep2
Ø H1975_unst_Rep3
Ø H1975_TGFß_Rep1
Ø H1975_TGFß_Rep2
Ø H1975_TGFß_Rep3
Ø H838_unst_Rep1
Ø H838_unst_Rep2
Ø H838_unst_Rep3
Ø H838_TGFß_Rep1
Ø H838_TGFß_Rep2
Ø H838_TGFß_Rep3
Comparing
2 Cell lines
Comparing
4 Stimulations
Comparing
12 Biological Replicates
Comparing
24 Technical Replicates

Figure 1: **Experimental design of the benchmark data set.** For both NSCLC cell lines H1975 and H838 a total of six biological replicates were seeded and cultivated. For the resulting analysis design created by *MSPypeline* (following the naming convention of the individual samples), this implies that the two cell lines constitute level 0 with each group comprising six samples. Three of the replicates from each cell line were stimulated with TGFβ and three replicates remained unstimulated. This treatment of each cell line represents the next higher level 1 in the analysis design that is subdividing the two cell line groups into four specified subsets. The biological replicates of each cell line and treatment combination account for level 2. Here the four groups are subdivided into twelve single samples. When performing data analysis with *MSPypeline*, the selected level of analysis design determines which groups will be compared. Level 3, which is indicating the technical replicates that have been measured from each sample, will be averaged in most of the analyses, leading to the omission of level 3.

References

(*1*) Hughes, C. S., Moggridge, S., Muller, T., Sorensen, P. H., Morin, G. B., and Krijgsveld, J. (2019). Single-pot, solid-phase-enhanced sample preparation for proteomics experiments. *Nat Protoc 14*, 68–85.

(*2*) Perez-Riverol, Y. et al. (2019). The PRIDE database and related tools and resources in 2019: improving support for quantification data. *Nucleic Acids Res 47*, D442–D450.
